# A Test of Activated Carbon and Soil Seed Enhancements for Improved Sub-Shrub and Grass Seedling Survival With and Without Herbicide Application

**DOI:** 10.3390/plants13213074

**Published:** 2024-11-01

**Authors:** Lauren N. Svejcar, Trace E. Martyn, Hayley R. Edlund, Kirk W. Davies

**Affiliations:** 1 Eastern Oregon Agricultural Research Center, United States Department of Agriculture-Agricultural Research Service, Burns, OR 97720, USA; hayleyedlund@outlook.com (H.R.E.); kirk.davies@usda.gov (K.W.D.); 2 Eastern Oregon Agricultural Research Center, Oregon State University, Union, OR 97883, USA; trace.martyn@oregonstate.edu; 3 National Park Service, Yellowstone, WY 82190, USA

**Keywords:** bluebunch wheatgrass, herbicide protection, krascheninnikovia lanata, pseudoroegneria spicata, seed enhancement technology, winterfat

## Abstract

Re-establishing native plants while controlling invasive species is a challenge for many dryland restoration efforts globally. Invasive plants often create highly competitive environments so controlling them is necessary for effective establishment of native species. In the sagebrush steppe of the United States, invasive annual grasses are commonly controlled with herbicide treatments. However, the same herbicides that control invasive annual grasses also impact the native species being planted. As such, carbon-based seed technologies to protect native seeds from herbicide applications are being trialed. In addition to controlling invasive species, ensuring good seed-to-soil contact is important for effective establishment of native species. In this grow room study, we explored the impact of different seed ameliorations when no herbicide was applied and when herbicide was applied. We selected two native species that are important to the sagebrush steppe for this study—the sub-shrub *Krascheninnikovia lanata* and the perennial bunchgrass *Pseudoroegneria spicata*—and used three different seed ameliorations—seed pelleting with local soil alone, local soil plus activated carbon and activated carbon alone—to ensure both greater seed-to-soil contact and protection against herbicides. Shoot and root biomass data were collected eight weeks after planting. We found that when herbicide was not applied, *K. lanata* had the strongest response to the soil alone amelioration, while *P. spicata* had the strongest response to the activated carbon alone amelioration. However, when herbicide was applied, *K. lanata* performed best with the soil plus activated carbon treatments, with an average 1500% increase in biomass, while *P. spicata* performed best with the activated carbon alone treatments, with an over 4000% increase in biomass, relative to bare seed. The results from our study indicate that there is a positive effect of local soils and activated carbon as seed ameliorations, and further testing in the field is needed to understand how these ameliorations might perform in actual restoration scenarios.

## 1. Introduction

Degraded dryland ecosystems are often faced with major challenges in re-establishing native species [1,2]. In dryland ecosystems such as the sagebrush (*Artemisia* spp.) steppe, the invasion of exotic annual grasses is creating unprecedented rates of degradation [3,4,5,6,7,8]. Areas of sagebrush steppe historically dominated by perennial species are converting to annual grasslands at a rate of approximately 200,000 ha per year [3]. Establishing native perennial sagebrush steppe species in exotic annual dominated systems is needed immediately to reduce ecological risks, such as species extinction due to habitat loss, and economic risks to land managers [9,10,11]. However, restoration of these degraded areas is challenging, as land managers must simultaneously control exotic annual grasses and successfully establish native perennial species before exotic annual grasses re-establish [9,12].

One of the challenges to establishing native perennial species is ensuring good seed-to-soil contact. Good seed-to-soil contact can help increase consistent moisture around the seed and improve the establishment of some seeded species, bunchgrasses in particular [13,14]. One method for achieving good seed-to-soil contact is through drill seeding, which demonstrates greater establishment potential of bunchgrasses relative to broadcast seeding through both space and time [15,16]. However, drill seeding is logistically impractical for many areas in the sagebrush steppe due to topography, specifically rocky ground and steep surfaces. As such, broadcast seeding is often the only technique that is logistically feasible for reseeding many degraded areas [12]. One possible means for increasing seed-to-soil contact in broadcast seeding is the amelioration of seeds through seed coatings or pelleting, wherein material coats the seed, but the seed can still be broadcast [17].

The amelioration of seeds for improved germination and establishment of seeded species has been utilized for centuries by indigenous communities in North America to overcome a wide range of limitations, including seed predation [18,19,20,21]. In the 1940s, the US government funded substantial research in the western US on the use of soil-based seed coatings following large-scale degradation due to overgrazing [22,23,24,25,26]. These efforts predominantly focused on non-native bunchgrasses, specifically crested wheatgrass (*Agropyron cristatum)*, and had variable success in the first few years following seeding. Long-term (7-year) evaluation of those seedings showed marked decreases in plant establishment, such that there was no benefit to pelleting [26]. The substantial decrease in emergent seedlings from bare seed and pelleted seed trialed in the early 1900s was in part attributed to competition from invasive species [26]. This suggests that providing control from competing invasive exotic species will be a priority in many areas.

Soil active herbicides, such as pre-emergent chemical methods for controlling invasive exotic species, are common globally [27] and widely used in the western US (including the sagebrush steppe) to control invasive annual grasses [28,29]. Recently, the pre-emergent herbicide Indaziflam (Esplanade 200 SC, Bayer CropScience, Monheim am Rhein, Germany) was approved for use in many areas of the sagebrush steppe and has demonstrated high efficacy in controlling exotic annual grasses [30,31,32,33,34,35]. However, native species’ seeds and seedlings can be inhibited by the same herbicides applied to control exotic annual grasses [35,36,37,38]. An emerging method in ecological restoration for protecting planted native species’ seeds and seedlings from herbicides is creating a carbon-based seed technology, wherein activated carbon surrounds the seeds [36,39,40]. Activated carbon is used to adsorb and deactivate herbicides in crop settings [41]. Using native seed mixed into protective pods for restoration and simultaneously applying herbicide treatment can help protect native seeds and reduce competition from invasive annual grasses [39,42]. While carbon-based seed technologies may help mitigate the impacts of herbicides, there is still a need to ensure seed-to-soil contact to alleviate fluctuations in moisture for improved establishment of seeded species [37].

Activated carbon is the key ingredient in seed technologies focusing on reduction in herbicide toxicity that have been studied to date. However, a range of other ingredients that act as binders (e.g., Selvol-205; [36,37]) or serve other functions like controlling pathogens via a fungicide (e.g., Captan 900 WG; [43]) or increasing nutrient content around the seed (e.g., worm castings and compost; [36]) are often added to these technologies. Sometimes, the combination of materials in seed technologies and the amount of material around seeds relative to the size of seeds can create a physical barrier for seedling emergence [40,42]. Given that some seed technologies can limit emergence, it is crucial to test seed technologies with and without herbicides to determine whether seed germination, emergence and establishment are limited by the technology or herbicides.

In this experiment, we considered how broadcast seeding efforts could be improved with seed coatings to ensure seeds have increased seed-to-soil contact and the impacts of herbicide applications are mitigated. To accomplish this, we tested pelleting of seeds with activated carbon, local soil and local soil plus activated carbon on two common species in the sagebrush steppe: a bunchgrass, bluebunch wheatgrass (*Pseudoroegneria spicata* (Pursh) A. Löve) and a half-shrub, winterfat (*Krascheninnikovia lanata* (Pursh) A. Meeuse and A. Smit). Both species are native to the sagebrush steppe and are commonly used in restoration because of their wide distributions and importance as forage species for wildlife and livestock [44,45]. While *P. spicata* has been repeatedly tested with carbon-based seed technologies [36,37,39,42], no carbon-based seed testing has been performed for *K. lanata*, and the use of soil as a seed technology has yet to be tested for either species. Our aim was to understand the impact of seed technology on these species. Specifically, our research questions were as follows:

1. When no herbicide is applied, how do the treatments (soil alone, activated carbon alone and soil plus activated carbon) affect shoot and root biomass in our two species?
2. When herbicide is applied, how do the treatments affect shoot and root biomass?

To address these questions, we set up a grow room study and applied seed technologies and herbicide treatments in a full factorial design.

## 2. Results

### 2.1. No Herbicide Applied

**Figure 1:**
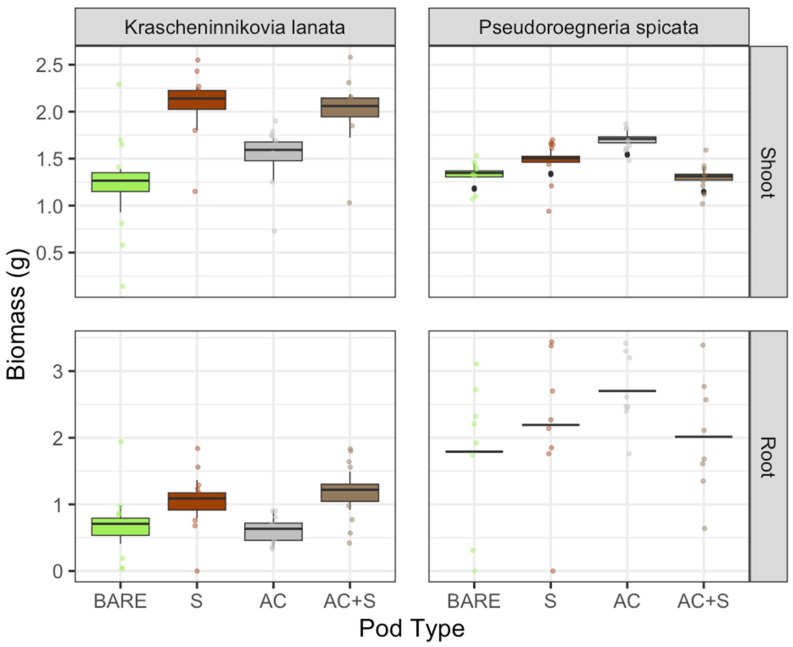
No herbicide applied: Root and shoot biomass for *Krascheninnikovia lanata* and *Pseudoroegneria spicata* with four different seed pod treatments (BARE = bare seed (control), S = soil alone, AC = activated carbon alone, AC + S = soil plus activated carbon) when herbicide was not applied. Boxplots are the model-predicted outcomes, and points are the actual raw values.

Shoot and root biomass within a species responded similarly to the pod treatments for each species when no herbicide was applied. *Krascheninnikovia lanata* mean shoot biomass increased by nearly 50% in both of the pod treatments with soil (soil alone and soil plus activated carbon) and was significantly different from bare seed (Figure 1). Similarly, *K. lanata* mean root biomass increased by over 70% with the soil alone pod treatment. However, mean root biomass was not statistically different from bare seed or the other seed pod treatments.

For *P. spicata*, we found that activated carbon alone had significantly greater shoot biomass compared to bare seed and soil alone by approximately 20%. However, with soil plus activated carbon and soil alone treatments, we saw significantly less shoot biomass compared to the activated carbon alone treatment (Figure 1). Mean root biomass demonstrated a similar pattern to shoot biomass for *P. spicata*. We saw an increase in root biomass with the activated carbon alone treatment compared to all other treatments. However, there were no statistically significant differences in root biomass for *P. spicata* across any of the treatments.

### 2.2. Herbicide Applied

**Figure 2:**
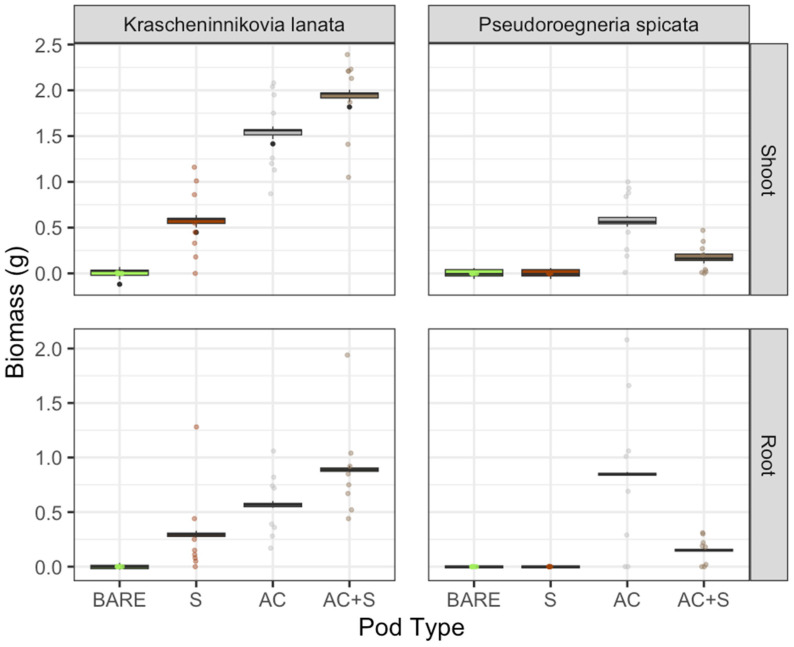
Herbicide applied: Root and shoot biomass for *Krascheninnikovia lanata* and *Pseudoroegneria spicata* with four different seed pod treatments (BARE = bare seed (control), S = soil alone, AC = activated carbon alone, AC + S = soil plus activated carbon). Boxplots are the model-predicted outcomes, and points are the actual raw values.

Addressing our second research question regarding the impact of pod treatments on biomass when herbicide was applied, we saw different patterns of biomass response for *K. lanata* but not *P. spicata*. For *K. lanata* shoot biomass, we found that all pod treatments had higher biomass compared to bare seed, with the soil plus activated carbon treatments performing best, with an average 1500% greater biomass. The soil plus activated carbon treatments were not significantly different from activated carbon alone treatments for *K. lanata* shoot biomass but were significantly different from the soil alone treatment (Figure 2). The soil alone treatment was also significantly different from bare seed, with 450% greater biomass. For *K. lanata* root biomass, we found a similar pattern to *K. lanata* shoot biomass when herbicide was applied. Root biomass was significantly greater with both activated carbon alone treatments compared to bare seed (which had an average biomass of 0.0 g). The soil plus activated carbon treatments were also significantly different from the soil alone treatment. The soil alone treatment was not significantly different from bare seed. For *P. spicata* plants, shoot and root biomass was significantly greater compared to the bare seed, with the activated carbon alone treatment resulting in over 4000% greater biomass. The activated carbon alone treatments also had significantly more biomass in roots and shoots than the soil plus activated carbon treatments (Figure 2).

## 3. Discussion

In our study, we found that the use of seed treatments provided a benefit to both *P. spicata* and *K. lanata* species when no herbicide was applied. *Pseudoroegneria spicata* had a stronger positive response to the activated carbon alone than to the soil alone and soil plus activated carbon treatments. Activated carbon has a high surface area and a high degree of surface reactivity [46,47], which may have influenced the moisture levels around seeds. Some laboratory trials of activated carbon seed technologies showed increased emergence and densities from pelleted formulations but comparable biomass when herbicide was not applied [42]. However, other studies demonstrated lower levels of biomass and mean seedling emergence from seed technologies relative to bare seed when no herbicide was applied [43]. The variation in responses seen in our study and other laboratory studies of activated carbon seed technologies could be due to variations in the material composition of seed technologies, such as the addition of different types and quantities of binders (e.g., Selvol, clays). Concerted experimental testing with individual materials for seed technologies (e.g., without the use of binders) and with diverse material sources, such as different types of activated carbons, are needed to better understand the various impacts that these materials have on seed germination and seedling growth. Similarly, the leaching and concentrations of herbicides in laboratory studies are likely not representative of field leaching and concentrations [38,48,49]. As such, it is critical to test these technologies in the field with varying herbicide applications.

*Pseudoroegneria spicata* is the most extensively tested species in dryland restoration studies with carbon-based seed technologies [36,37,39,42,50,51,52]. However, this is the first study to test the effects of carbon-based seed technologies on *K. lanata*. *Krascheninnikovia lanata* had an inverse response to that of *P. spicata*, with the soil and soil plus carbon additions demonstrating the strongest positive trends with and without herbicide application. Multiple factors might contribute to the positive response of *K. lanata* to soil. First, *K. lanata* seeds have a fuzzy texture due to fluffy bracts [45,53,54,55]; therefore, soil amendments to seed may increase the moisture content immediately surrounding the seed. Another possibility is that there may be a relationship between *K. lanata* seeds and soil microbial communities. There is currently no clear consensus on whether soil microbial associations, such as arbuscular mycorrhizal fungi (AMF), provide a benefit to the germination and growth of diverse species [56]. Further, it is likely that the microbial associations will be species-specific. For example, it has been shown that inoculating big sagebrush seedlings with native AMF improves seedling survival when transplanted [57] and under drought stress [58]. Relationships between seeds and soil microbial associations are an understudied component of the sagebrush steppe ecosystem, and more studies are needed to better understand these connections [56].

When herbicide was applied, both species performed best with an amendment that had some activated carbon. Similar to when no herbicide was applied, *P. spicata* performed best with the carbon only amendment. This result agrees with previous research that found a positive effect of carbon in providing some level of protection for *P. spicata* when different pre-emergent herbicides were applied in grow room conditions [36,42]. However, *K. lanata* had the greatest biomass of shoots and roots with the soil plus carbon amendment when herbicide was applied, followed by the carbon only amendment. The strong impact of soil on *K. lanata* growth was apparent with and without herbicide application. Further research into the mechanism of this growth response would provide insight into how soil amendments could be used to optimize seeding success. While the results of our study inform future lines of research, it was conducted in a grow room setting, which has limitations in determining the realized success of seed technologies for restoration applications. Field studies in a wide range of climatic and edaphic contexts will be necessary to determine where and what types of seed technologies will be appropriate for land managers in restoration efforts.

Research in the early 20th century that utilized soil as an amendment to seeds was conducted on grass species, with a specific focus on non-native species like crested wheatgrass [22,24,59]. The results from these studies showed generally low success with the soil amendments, which was attributed to issues with mechanical compression of the seed technology in the production process. We created the seed technologies in this experiment by hand; therefore, seed compression was not an issue. However, our results with *P. spicata* are similar to those from the early 20th century, in that soil did not provide a particularly large benefit. However, the strong response of *K. lanata* to soil amendments demonstrates that the benefit of a particular amendment may be highly species-specific. Our results of differential responses of the two species to seed technologies are not surprising, as one seed technology formulation will likely not be ideal for all situations and species, and they demonstrate a need for broad testing of species, the plant materials of these species and soil amendment material composition in a wide range of environmental contexts.

## 4. Conclusions

The sagebrush steppe is at a tipping point for species conservation, with invasive species spreading to sites that were historically at low risk [60]. Increasing the pressure on native sagebrush steppe plant communities is creating a greater need for effective solutions to restoring native perennial species. Alleviating competition from exotic annual grasses is critical for effectively restoring native perennial vegetation [12,61,62], and as such, solutions to simultaneously control exotic annual grasses and establish native perennial vegetation are desperately needed for the conservation of the sagebrush steppe ecosystem [9]. Finding effective methods for restoring perennial vegetation following the control of exotic annual grasses is critically important to the long-term potential of a site. Our study provides evidence for two potential formulations of seed technologies that may help alleviate the impacts of herbicides and improve seed-to-soil contact. However, this study was conducted in a laboratory setting and did not have any invasive species planted. Further testing of these seed treatments in diverse environmental and climatic contexts with varying herbicide applications, varying densities of invasive species present and diverse restoration species combinations are needed to improve our ability to convert annual-grass-dominated areas back to perennial vegetation.

## 5. Materials and Methods

The experiment was conducted in a grow room at the Eastern Oregon Agricultural Research Center in Burns, OR, USA. In our experiment, we used soil collected in eastern Oregon from the Northern Great Basin Experimental Range (43.489770, −119.710736), which is located in the sagebrush steppe ecosystem. The soil was a Gochea sandy loam (USDA NRCS 2019) and was collected at the end of summer to ensure it was predominantly dry. The top 10 cm of soil was collected and used both for creating the pellets and filling the pots. We sifted the soil to exclude particles above 1.4 mm and dried the soil in ovens at 65 °C for 48 h to remove any residual moisture. For our study, we purchased seeds from a commercial grower (Granite Seed Company, Lehi, UT, USA).

### 5.1. Project Design

The study was set up as a complete randomized block design: 2 species × 2 herbicide treatments × 4 seed treatments × 8 blocks, with each block having only one replicate for each species x treatment combination for a total of 128 pots. We had two herbicide treatments: indaziflam applied and indaziflam not applied. We had four seed treatments: bare seed (control), seeds incorporated into activated carbon (activated carbon alone), seeds incorporated into a soil (soil alone) and seeds incorporated into a combination of activated carbon and soil (soil plus activated carbon) (Table 1). The activated carbon used was Nuchar^®^, which is a chemically activated wood-based product (Ingevity; North Charleston, SC, USA). The seed pod ingredients were mixed by hand at ratios per gram of seed (Table 1); therefore, the relative coverage of material was similar for the large seeded *P. spicata* and the smaller seeded *K. lanata*, following the methodological approach of Baughman et al. [50]. We then stirred seeds into the mixture, and seed pods were hand-rolled into small balls 2.5 cm in diameter. *Krascheninnikovia lanata* seeds were not debearded prior to mixing them into seed pods.

We filled each 12.7 cm square plastic pot with 2.33 kg dry soil (surface soil area of 177.8 cm^2^). Five pods or bare seeds were placed on the soil surface in each pot. We ensured that each pot contained 30 pure live seeds of *K. lanata* or 33 pure live seeds of *P. spicata*, as informed by the seed production company and verified with in-house germination trials. We assigned treatments to each pot within each block using a random number generator. Pots were irrigated with 400 mL of water 2 h prior to herbicide spraying and 100 mL 12 h after herbicide spraying, which was 75% of the water-holding capacity of the soil (*w*/*w*). Pots that were not sprayed with herbicide also received a total of 500 mL for 75% water-holding capacity in the same time period. We monitored soil moisture every other day by weight for the first two weeks to ensure surface soils did not dry out. After the first two weeks, we monitored soil moisture once a week by weight, and soil moisture was maintained at 75% water-holding capacity of the soil until the end of the study. In total, we ran the study for 8 weeks.

Indaziflam is a pre-emergent, cellulose biosynthesis-inhibiting herbicide that was recently approved for use in non-cropland settings in multiple western US states that could be effective in controlling numerous invasive winter annual grass species of high concern [33]. We applied Indaziflam (Esplanade 200 SC, Bayer CropScience, Monheim am Rhein, Germany) at a rate of 46.7 g of active ingredient per hectare. This was the lowest rate found to be effective, following the results from Clenet et al. (2019). Herbicide-treated pots received 7.5 g of herbicide spray mixture per pot at 28 cm above the pot using a spray bottle. PlatinumLED P1200 lights (PlatinumLED, Kailua, HI, USA) were set 61 cm above pots and provided 8 h of visible spectrum light where we had a quantum flux of 800 µmol photons m^−2^ s^−1^ measured from the top of the container soil surface per day [63].

### 5.2. Measurements

Seedling emergence and height were measured every other day for the first two weeks of the study (Figure S1). Final seedling survival counts, above-ground biomass and below-ground biomass were collected 8 weeks after the start of the study. Final above- and below-ground biomass samples were dried in a plant oven at 65 °C for 48 h and weighed.

### 5.3. Statistical Analysis

To address our first research question concerning the impact of seed treatments on shoot and root biomass without herbicide application, we used a regression model fitting approach. We centralized our data first by subtracting the biomass measurements for each of the treatments for each species from the average bare seed value (i.e., control) for each species. This means that a positive value was an increase in biomass when the seed pod treatment was applied, and a negative value was a decrease in biomass. We then used these centered biomass values as response data for a mixed-effects linear model using a Gaussian distribution (glmmTMB, v 1.1.7; [64]). In our model, the block was used as a random effect, and the seed treatment was used as a fixed effect. After fitting the model, we then used the model to output potential mean outcomes of the seed treatments for each species (emmeans, v 1.10.2; [65]). We then performed Tukey’s post hoc analysis to determine significant differences between the different seed treatments (lmerTest, v 3.1-3; [66]). For clarity in presenting the results, we added these model outcomes back to the bare seed average value to present biomass in absolute values instead of differences from bare seed.

We used the same regression approach as above to address our second research question on the impacts of the seed treatments on biomass when herbicide was applied. We fit the mixed-effects model for each species using the centralized biomass response values and estimated the mean predicted outcomes of the fit models.

## Figures and Tables

**Table 1:** Quantities of activated carbon, soil and water for each species.

Species	Treatment	Activated Carbon (g)	Soil (g)	Water (g)
*Pseudoroegneria spicata*	Carbon coated	3.77	-	8.89
	Carbon and soil coated	2.07	8.74	6.19
	Soil coated	-	14.19	4.90
*Krascheninnikovia lanata*	Carbon coated	2.23	-	7.14
	Carbon and soil coated	1.25	5.26	7.89
	Soil coated	-	9.78	7.42

## Data Availability

Data will be made available upon acceptance of the article.

## Supplementary Materials

The following supporting information can be downloaded at: https://www.mdpi.com/article/10.3390/plants13213074/s1, Figure S1: Seedling counts and height. Ref. [67] was cited in the Supplementary Materials.
