# Supplementary material for: A Test of Activated Carbon and Soil Seed Enhancements for Improved Sub-Shrub and Grass Seedling Survival With and Without Herbicide Application
Source: Plants (Basel). 2024 Nov 1;13(21):3074. doi: 10.3390/plants13213074 (PMC11548513; doi:10.3390/plants13213074)
Supplement: Supplementary file 1 [file plants-13-03074-s001.zip › plants-3165337-supplementary.pdf]

Supplementary Materials

Svejcar et al. A Test of Activated Carbon and Soil Seed Enhancements for Improved Sub-Shrub and Grass Seedling Survival With and Without Herbicide Application. Plants.

Fig. S1a. Seedling counts. Seedling counts were made every two days for the first two weeks of the study and then periodically thereafter until the final count 8 weeks (56 days) after the start of the study. Counts of living seedlings were made for *Krascheninnikovia lanata* (KRLA) and *Pseudoroegneria spicata* (PSSP) with four different seed pod treatments (BARE = bare seed (control), S = soil alone, AC = activated carbon alone, AC + S = soil plus activated carbon) when herbicide was applied (H) and was not applied (NH). Points are raw data counts and lines are fitted with a loess t-based approximation (ggplot2, v 3.5.1, [1]).

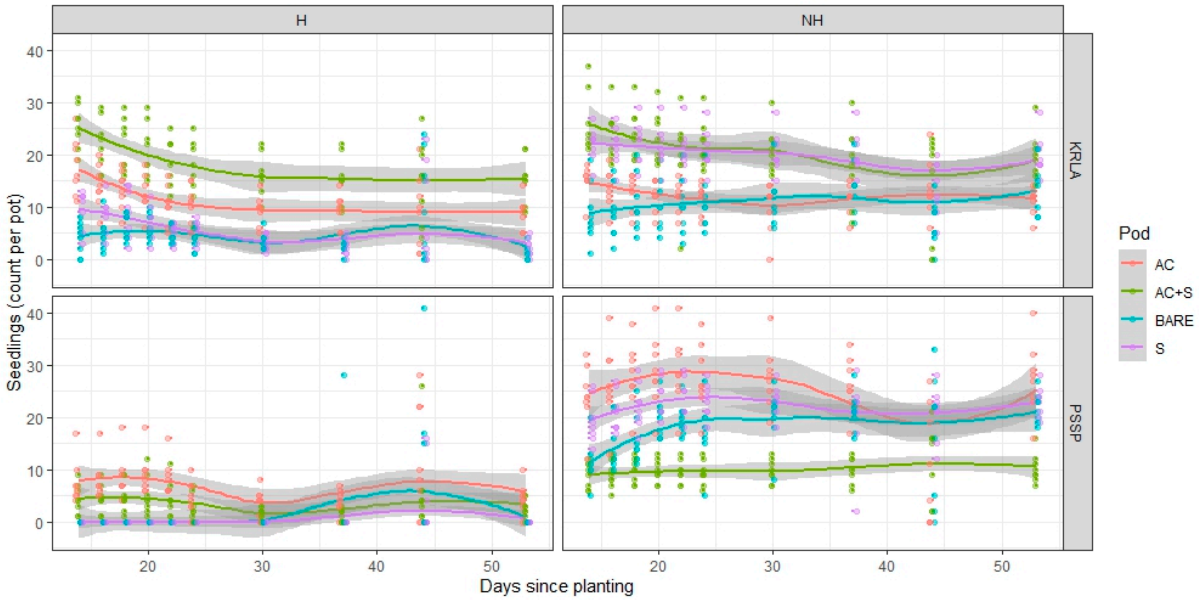

Fig. S1b. Seedling height. Seedling maximum height per pot was made every two days for the first two weeks of the study and then periodically thereafter until the final count 8 weeks (56 days) after the start of the study. Height measurements of living seedlings were made for *Krascheninnikovia lanata* (KRLA) and *Pseudoroegneria spicata* (PSSP) with four different seed pod treatments (BARE = bare seed (control), S = soil alone, AC = activated carbon alone, AC + S = soil plus activated carbon) when herbicide was applied (H) and was not applied (NH). Points are raw data heights and lines are fitted with a loess t-based approximation (ggplot2, v 3.5.1, [1]).

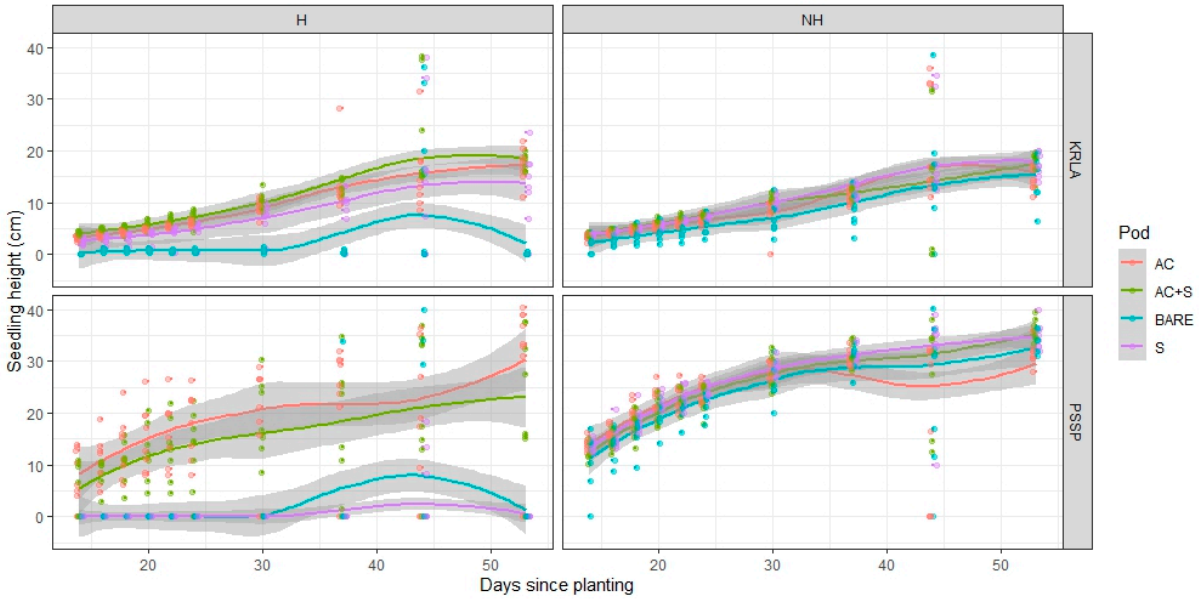

Reference

1. Wickham, H. *Ggplot2*; Springer Science+Business Media, LLC: New York, NY, 2016; ISBN 978-3-319-24275-0.
